# Peruvian Maca: Two Scientific Names *Lepidium Meyenii* Walpers and *Lepidium Peruvianum* Chacon – Are They Phytochemically-Synonymous?

**Published:** 2015-03

**Authors:** Henry O. Meissner, Alina Mscisz, Bogdan Kedzia, Pawel Pisulewski, Ewa Piatkowska

**Affiliations:** 1 Faculty of Health Studies, Charles Sturt University & Therapeutic Research, TTD International Pty Ltd, 39 Leopard Ave., Elanora, QLD 4221, Australia;; 2 Research Institute of Medicinal Plants, 27 Libelta St., 61-707 Poznan, Poland;; 3 Faculty of Food Technology, University of Agriculture, Krakow, 30-149 Krakow, Poland

**Keywords:** Glucosinolytes, Hypocotyl, Lepidium meyenii, Lepidium peruvianum, Methoxy flavon, Peruvian Maca

## Abstract

Using Liquid Chromatography-Mass Spectrometry (LCMS) , profiles of the two isotypes labelled under the same common name Maca deposited in the Medicinal Plant Herbarium, in Australia and Poland, but identified under two different scientific names *Lepidium meyenii* Walpers *(L. meyenii)* and *Lepidium peruvianum* Chacon *(L. peruvianum)* are presented. The two isotypes correspond to two holotypes of Peruvian medicinal herb known under the same common name “Maca”, as originally deposited in the Herbarium of San Marcos University in Lima, Peru dated back to 1843 and 1990 respectively.

The results demonstrate distinct differences in taxonomy, visual appearance, phytochemical profiles and DNA sequences of the two researched Maca isotypes, suggesting that the two Maca specimens are dissimilar and formal use of the term “*synonymous*” to *L. meyenii* and *L. peruvianum* may be misleading. On the basis of presented results the scientific name *L. meyenii*, used since 1843 up-today for cultivated Peruvian Maca by numerous reference sources worldwide, including Regulatory Bodies in the USA, EU, Australia and most lately in China, appears to be used in error and should be formally revised.

It is concluded, that the isotype of cultivated Peruvian Maca labelled under its scientific name *Lepidium peruvianum* Chacon, provides all the characteristics peculiar to this historically-documented herb grown in Andean highlands, which may be linked to its traditional use and accepted functionality, confirmed in recent clinical study to be relevant to its present day use for expected dietary, therapeutic and health benefits.

## INTRODUCTION

Peruvian Maca – a centuries-old South American medicinal herb restricted in distribution to high Andean plateaus above 4,000 m a.s.l. – has been traditionally used by indigenous Peruvians as a vital dietary supplement and an important staple food component in their diet (1-6). This ancient plant cultivated by natives since Incas times is believed to have favorable effects on energy and mood, fertility, improving sexual desire and decreasing anxiety, to mention a few. During the last 50 years or so, several clinical and laboratory studies have confirmed the relevance of specific health benefits linked to its traditional use (5-11).

Peruvian Maca is known internationally under its scientific name *Lepidium meyenii* Walpers (12) - the name in use since 1843 when German botanist Gerhard Walpers deposited the first specimen-holotype of this plant in the Herbarium of San Marcos University (SMU) in Lima. In 1960, Peruvian botanist Gloria Chacon-Roldan (13) has described Maca under the alternative name – *Lepidium sp.,* but it was not until the 1990, when she deposited in the same Herbarium, an identically-termed holotype of Maca (14) under the new scientific name *Lepidium peruvianum* Chacon (15). Chacon (5) claimed that the *L. meyenii* represents a wild growing Maca plant, while *L. peruvianum* represents cultivated Peruvian Maca linked to its Incas origin. Since then, numerous dietary supplements and functional Maca products distributed on local and international markets as Peruvian Maca are identified on the label under scientific name, either as *L. meyenii* or *L. peruvianum*.

Maca is the only member of *Brassicaceae* family and its genus *Lepidium* with a fleshy, starch-containing subterranean storage part (called tuberous root or hypocotyl), which is usually formed after being fused with the taproot, resulting in a turnip- or reddish-like tuberous body (16).

Several groups of biologically active constituents have been identified in edible underground part of cultivated Peruvian Maca (5, 7, 9, 17, 18). One group of compounds described by Johns (2) present in high concentrations, was classified as aromatic Glucosinolates (benzyl and p-methoxbenzyl glucosinolates in particular) and their isothiocyanate derivatives. The presence of these compounds in cultivated Maca and various plants from the *Brassicaceae* family have been confirmed by several authors (3, 19, 20-24). It could be stipulated that Glucosinolates profiles in Peruvian Maca could provide a “Fingerprint” helping to identify wild-grown and cultivated Maca plants. There is no information available in the literature as to Glucosinolates profiles in Maca holotypes deposited in the SMU Herbarium in Lima by Walpers in 1843 and Chacon in 1990, nor in any isotype of Maca specimen deposited in Herbaria overseas (5) and labelled Maca.

Therefore, in this paper, based on comprehensive Glucosinolate profiles in both aerial and subterranean parts, a comparison of phytochemical characteristics in specimens of the two isotypes deposited in the Australian and Polish Herbarium of Medicinal Plants under the same common name “Maca” of Peruvian origin, but labelled under two different scientific names - *Lepidium meyenii* Walpers *(L. meyenii)* and *Lepidium peruvianum* Chacon *(L. peruvianum)* is presented.

## METHODS

### Material

The Herbarium at USM in Lima has not allowed tampering with holotypes of Maca specimens originally deposited by Walpers in 1843 as *L. meyenii,* nor any available holotype of *L. peruvianum* as deposited in the same Herbarium by Chacon (14). Therefore, in order to obtain samples of both plants for analytical testing, between 2000 and 2004, several expeditions led by an experienced botanist, have explored the very region of Andes where the plant *L. meyenii* was reported as may still grow in the wild. The searching party eventually found and collected several specimens of *L. meyenii* which were prepared, as per requirements for deposition of isotypes in Herbarium.

Isotypes of *L. peruvianum* were collected from a field of cultivated Maca in Junin plateau, Department of Pasco (Huaraucaca), where Chacon has sourced the Maca holotype deposited in the USM Herbarium (Holotype #89129), the location, from where Maca was also sourced for previously reported clinical study (24).

The authenticity of *L. meyenii* and *L. peruvianum* isotypes was verified in the USM Herbarium in terms of taxonomic characteristics being similar and phenotypically conforming to the original voucher specimen deposited by Walpers back in 1843 and Chacon in 1990. In addition, Dr G. Chacon personally identified isotype of *L. peruvianum*, as the cultivated Peruvian Maca conforming to the characteristics of the original holotype she has deposited in 1990 in the USM Herbarium under the name “Maca” and similar to isotypes also sent earlier to Herbariums in Berlin, Germany, Berkeley, CA, USA and Zurich, Switzerland (5).

In 2005, isotypes of both specimens were sent from Peru to Australia and Poland and formally added to respective collections held in the Southern Cross University (SCU) Herbarium of Medicinal Plant in Lismore, Australia and in Herbarium of Medicinal Plants at the Research Institute of Medicinal Plants (RIMP) in Poznan, Poland. Sub-samples of both specimens *L.meyenii* and *L. peruvianum* were assessed phenotypically and used further in analytical procedures.

### Analytical Procedures

**Chromatographic (HPLC) spectra: *L. meyenii* and *L. peruvianum*:** The glucosinolate profiles in the aerial and subterranean parts were comparatively examined by HPLC method. The method described by Piacente *et al.* (25) and McClure (26) was adopted with modifications, so as to adjust it to internal laboratory conditions (27).

The HPLC resolutions were profiled on a long, polar to intermediate polarity gradient, suitable for examination of a broad range of phytochemical classes, thus providing clearer separation of glucosinolates and a more comprehensive comparison of selected sections of profiles with different compounds of interest. The common glucosinolate, Sinigrin (2-propenyl glucosinolate) derived from black mustard was used as a reference substance.

Plant specimens were prepared for analysis by HPLC as follows: the dried sub-samples of aerial or subterranean parts of each *L. peruvianum* and *L. meyenii* were ground in a Waring blender and extracted in methanol with 15 minutes sonication. The extracts were centrifuged at 3,500 rpm for 10 minutes and the supernatant sampled for analysis of Glucosinolates (as Benzyl glucosinolates and derivatives thereof) determined by HPLC using UV-Vis detection within the range 230-300 nm and between 2.1 and 4.0 min detection scan.

Comprehensive phytochemical profiling was performed on a Phenomenex Luna C18, 4.6 × 100 mm, 3 μm HPLC column (ARL-TM125). An Agilent 1100 HPLC was equipped with a PDA detector and coupled to an Agilent 1100 Mass Selective detector with an APCI interface. Detection was attained between 190–600 nm and APCI Pos, 150eV respectively. The mobile phase employed was A: water 0.05% TFA, B: Acetonitrile 0.05% TFA from 10% B - 95% B over 20 mins, flow rate 0.75 mL/min.

Specific glucosinolate analysis was performed on a Phenomenex Synergi 4 μm, 250mm × 4.6mm HPLC column (ARL-TM125). However, in this part of the study, the column was eluted isocratically with 0.05M KH_2_PO_4_ over 6 mins followed by a gradient elution to 0.05 M KH_2_PO_4_: ACN over 19 mins. The column oven temperature was 40°C with a flow rate of 1.0 mL/min and a 10 μL injection volume. Glucosinolates were detected at 225 nm.

The resultant HPLC spectra and resolution profiles of *L. meyenii* and *L. peruvianum* were then both used as “fingerprints” characterizing each analyzed subterranean part of Maca isotype. In one of several analytical HPLC separation cycles for subterranean parts of both isotypes, differences in resultant spectra were extended into 3D Maca spheroids.

**Preliminary DNA identification:** In order to extend HPLC to potential genetic profiling, an attempt has been made to compare the two isotypes from co-depositions in the Herbarium of Medicinal Plants located in the SCU Lismore, Australia and RIMP in Poznan, Poland, by applying routine DNA sequencing technique. Extraction of viable protein and isolated genomic DNA was performed on one gram of grated and pulverised under liquid nitrogen samples of dry subterranean parts of *L. meyenii* and *L. peruvianum* using procedure developed in Molecular Plant Breeding Laboratory, the Royal Melbourne Institute of Technology (RMIT). After confirmation of DNA presence amplification and DNA sequence detection was performed using a Hybaid thermocycler.

As there were no sequences available in Gene Bank for *Lepidium* species, the following available sequences belonging to *Lepidium draba* (28) were used: AY733091, AY733101, AY733096, AY733091.1 and AY733095. These sequences were applied as microsatellite markers and were obtained through use of Biomanager. Synthesized microsatellites with the above sequences were obtained from Gene Works Pty Ltd.

## RESULTS

**Figure 1:**
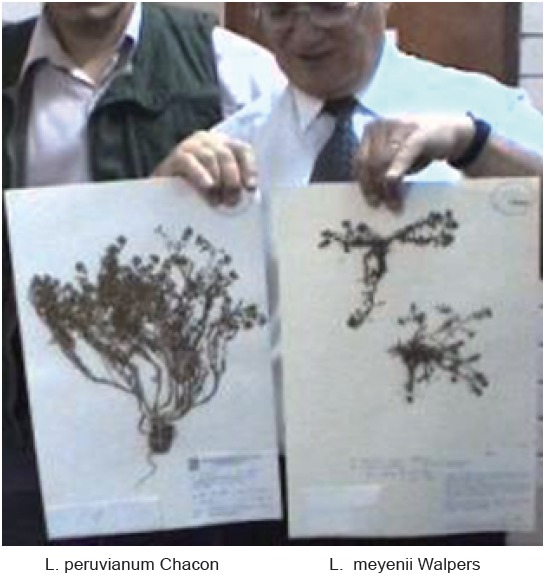
Original accession of the two holotypes of Macaplant kept in the Herbario del Museo, University of San Marcosin Lima, Peru, labelled “Maca” – *Lepidium peruvianum* Chacon (left) which shows distinctive phenotypical differences in leaves and tuberous root (hypocotyl), not present in Maca – *Lepidium meyenii* Walpers (right).

**Figure 2:**
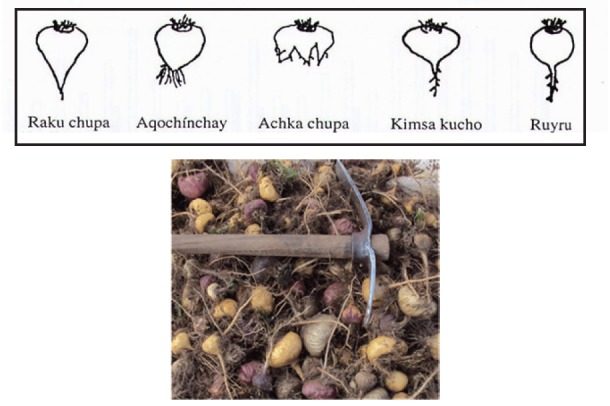
Basic shapes of hypocotyls in cultivated Maca according to the most commonly-observed in the Maca crop (upper box) in relation to hypocotyls observed in freshlyharvested crop of Peruvian Maca cultivated in Junin Region (photo – insert below) indicating that the most common shape can be identified after Echegaray (29) as “Kimsa kucho”.

After having an opportunity to access and personally inspect original depositions of the two Maca holotypes in Herbarium of Natural History USM in Lima, it appears, that the two Maca depositions indeed look differently (Figure 1). A distinctive oval tuberous root of *L. peruvianum* conformed to the shape identified in the literature (29, 30) as “Kimsa kucho” (Figure 2) while *L. meyenii* specimen had only a slightly thickened upper part of a tap root and a straight long root - without a distinctive tuber-like form along its shaft, which is a characteristic feature in cultivated Peruvian Maca - *L. peruvianum*.

### Aerial part

**Figure 3:**
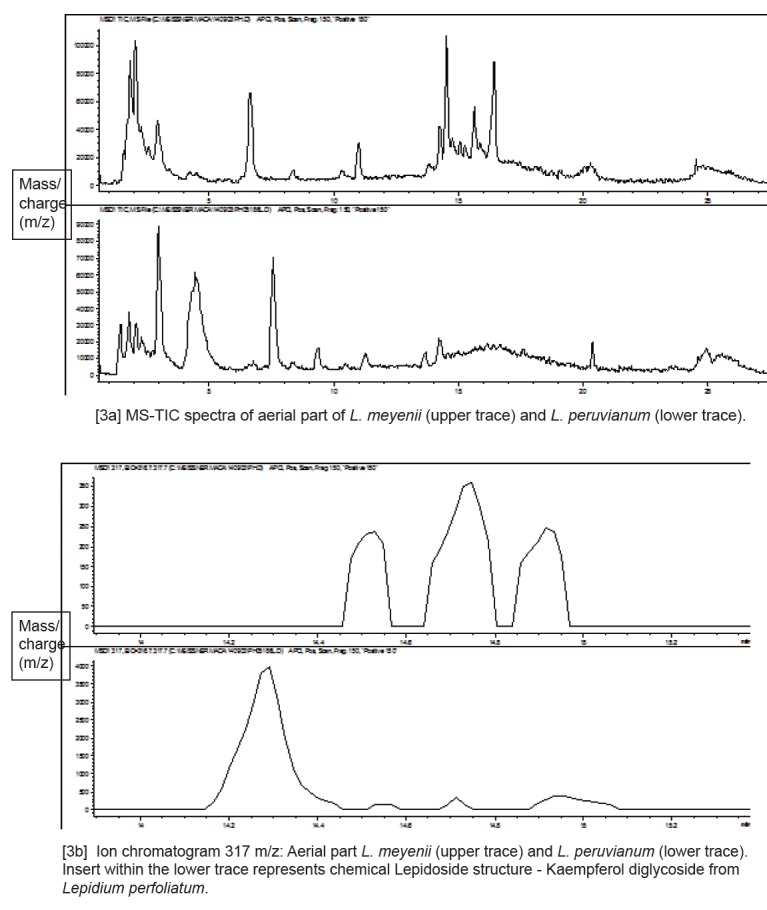
Comparison of MS-TIC spectra [1a] and MS fragmentation spectra of kaempferol and quercetin diglycosides [1b] in the aerial part of *L. meyenii* (upper traces) and *L. peruvianum* (lower traces).

**Figure 4:**
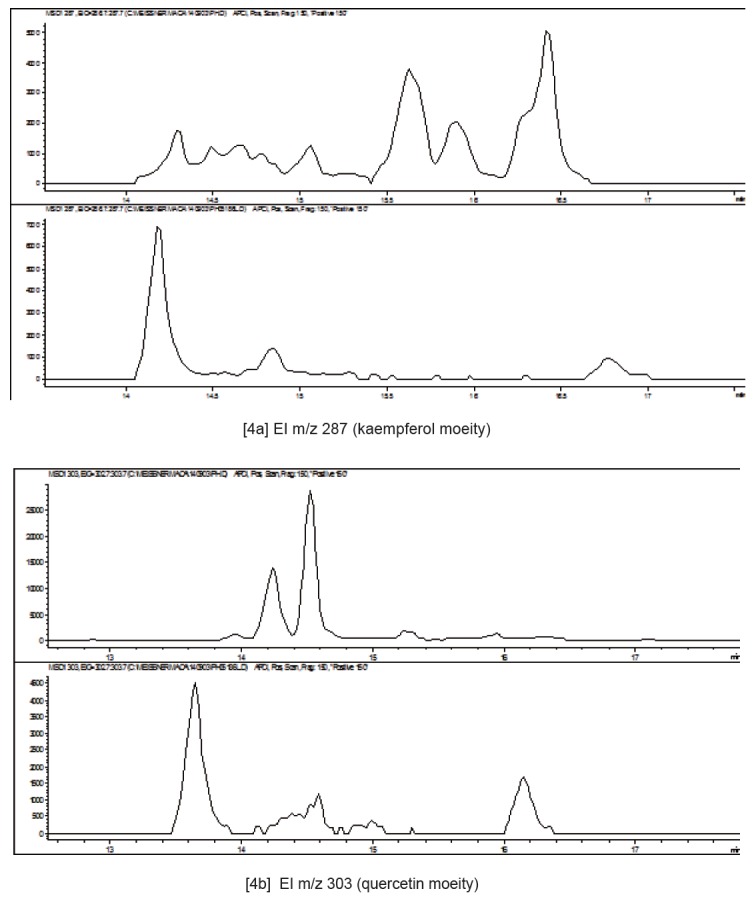
Comparison of Extracted Ion m/z 287 (kaempferol moeity) [2a] and m/z 303 (quercetin moeity) [2b] in aerial part of *L. meyenii* (upper traces) and *L. peruvianum* (bottom traces).

Glucosinolates have not been detected in the aerial parts of Maca. However, examination of the MS spectra for flavonoids compounds in the aerial part *L. meyenii* and *L. peruvianum* (Figure 3a) show some commonality of the both analyzed specimens, but also demonstrate an existence of distinctive differences in the m/z 317 extracted ion profiles (Figure 3b). Distinct flavonoid compounds present in *L. meyenii* (upper trace) are absent or present in trace quantities in *L. peruvianum* (lower trace), while very distinctive flavonoid compound in *L. peruvianum* was not detected in *L. meyenii.* Those differences between the two Maca isotypes were further confirmed (Figure 4) in comparison of Extracted Ion m/z 287 (Figure 4a) and m/z 303 (Figure 4b), representing kaempferol and quercetin moeity respectively.

**Figure 5:**
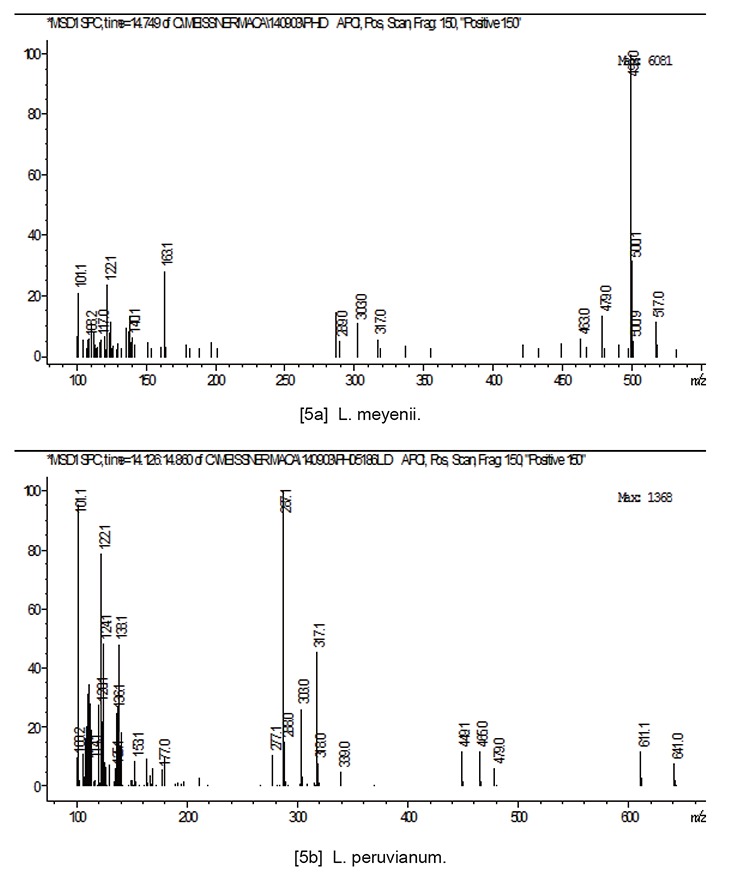
MS fragmentation spectrum characterising methoxy flavones in aerial part of *L. meyenii* (upper chart) and *L. peruvianum* (bottom chart).

Figure 5 shows the MS fragmentation spectrum pattern of the compounds present in aerial part of *L. meyenii* and *L. peruvianum*, co-eluted with another flavonoid. The proposed MS fragmentation pattern of this molecule is M+H 641, 479, 317, 287, which appears to be consistent with methoxy flavone diglycoside.

### Subterranean part

**Figure 6:**
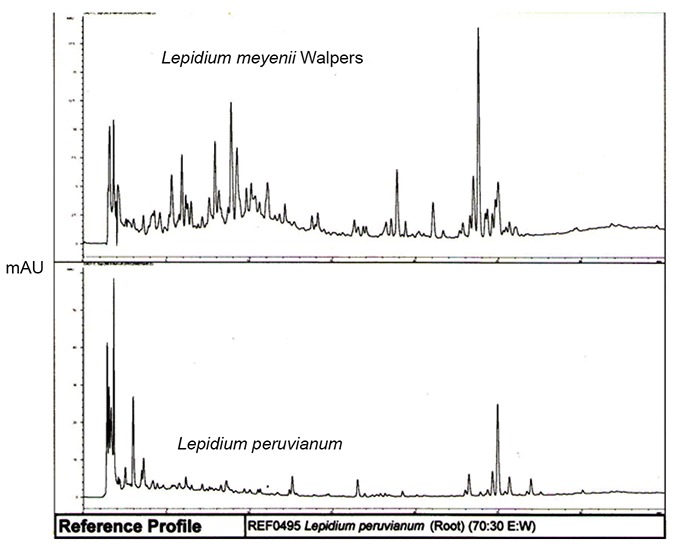
HPLC resolutions of subterranean parts in two Maca isotypes stored in the SCU Herbarium of Medicinal Plants, Lismore, Australia showing on substantial differences in traces of chromatograms of *Lepidium meyenii* Walpers specimen (upper trace), phenotypically representing a thin long mature root without visible hypocotyl formed and *Lepidium peruvianum* Chacon (bottom trace) – characteristic to cultivated Maca with well-developed and fully formed hypocotyl (for phenotypical differences refer to Figure 1).

**Figure 7:**
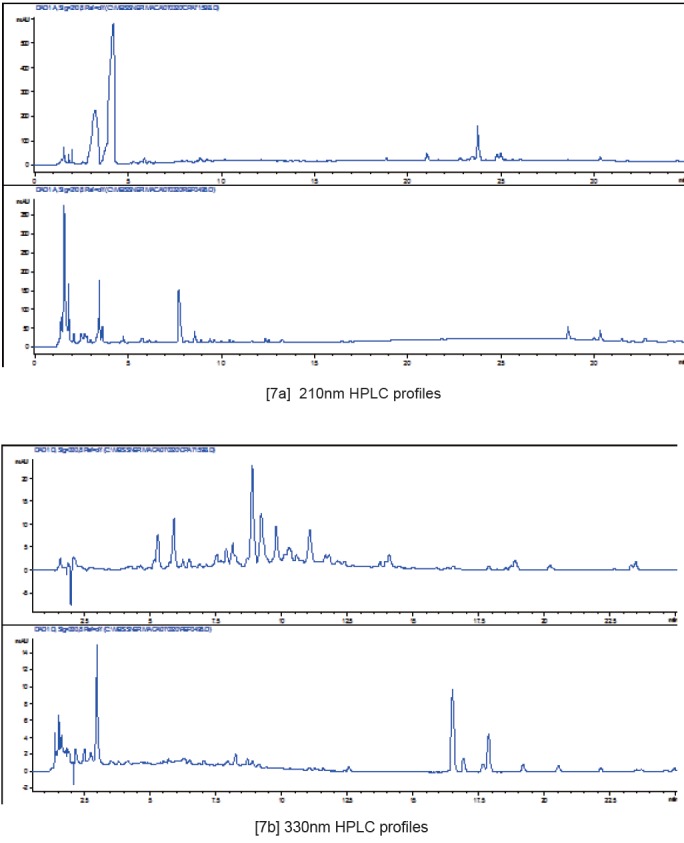
Comparison of 210nm [5a] and 330nm HPLC profiles [5b] of subterranean part: *L. meyenii* (upper traces) and *L. peruvianum* (lower traces) respectively.

Preliminary HPLC comparison of Glucosinolates spectra in the two Maca isotypes recorded back in 2007 after deposition of both specimens in the SCU Herbarium (Figure 6) indicated an existence of distinctive difference in the pattern of resolution peaks and overall shape of HPLC traces, which eventually led to more complex analysis of the aerial and subterranean parts of the isotypes as reported in this paper. Examining subterranean parts of each specimen by the same method as for aerial parts show, that as compared to *L. peruvianum,* the HPLC traces for subterranean part of *L. meyenii* have essentially different profile in terms of shape and retention time for the key components present, as observed on both 210nm and 330nm HPLC profiles in terms of phytochemical composition and abundance (Figure 7).

**Figure 8:**
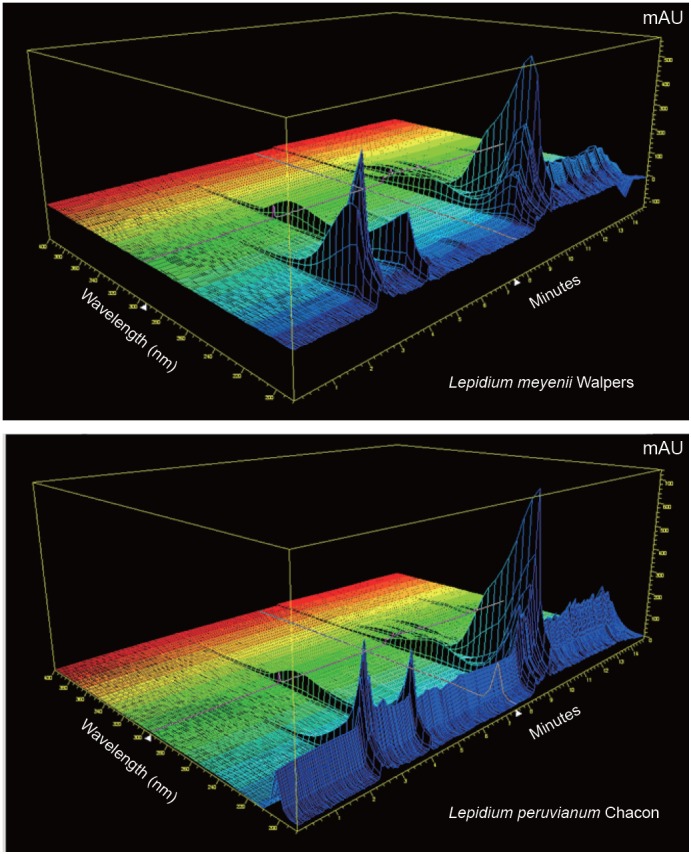
3D HPLC Resolutions: *L. meyenii* Walpers and *L. peruvianum* Chacon. Chromatographic profiles of the two Maca isotypes generated the use of 3D plot mode of HP Chemstation software from analysis of Glucosinolates selected as active compound characterizing the cultivated Peruvian Maca - *L. peruvianum*.

Similarly to the 2D resolutions depicted as HPLC traces, 3D spheroids of the two isotypes (Figure 8) further confirmed observation that the qualitative glucosinolates profiles do not share commonality in terms of 3D profiling of the two tested Maca isotypes.

The scope of sample collection for this study was restricted due to availability of the plant material for sampling at its source, therefore, results were not statistically processed and qualitative comparison of obtaining HPLC traces is presented only. With more material available for analysis and HPLC resolutions from multiple samples, with the use of appropriate integration software package(s), it will be possible to express observed obvious differences in HPLC traces statistically and determine a degree of significance in selected section(s) of traces (say between 2.1 and 4 min detection scan) for the two tested isotypes.

**Figure 9:**
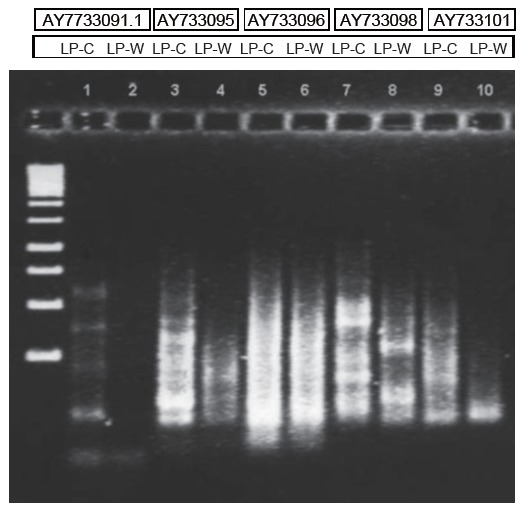
DNA extracted from dried Maca root *L. meyenii* Walpers (LP-W) and hypocotyls of *L. peruvianum* Chacon (LPC). Lanes numbered: 1, 3, 5, 7, 9 represent LP-C and lanes 2, 4, 6, 8, 10 represent LP-W. Semi-distinct lanes observed, particularly in lanes 7 and 8, may indicate an existence of genetic polymorphism.

Preliminary results from a test trial using DNA extraction from a dried root of *L. meyenii* Walpers (LP-W) and hypocotyl of *L. peruvianum* Chacon (LP-C) demonstrate that lines numbered 1, 3, 7 and 9 representing LP-C show more bands as compared to corresponding lines 2, 4, 8 and 10 with the use of the same primer (Figure 9), which may indicate an existence of genetic polymorphisms.

## DISCUSSION

For several years, Maca was officially labelled by Peruvian Ministry of Agriculture with scientific name *Lepidium meyenii* Walpers (31). Initially, Maca as the plant grown in its historically-documented location originating in Junin plateau was botanically characterized back in 1960 by Chacon (13), who labelled it with scientific name *Lepidium sp.* and categorizing it as an annual herbaceous plant with a tuberous root and the stem being almost acaule with strongly petiolate basal leaves. Contrary to Chacon (13), Brinckmann (32) considered Maca as herbaceous perennial plant, while Hermann and Bernet (33) classified Maca as a seed-propagated crop invariably behaving as herbaceous biennial plant in its native habitat.

The edible part of a mature Maca plant, which displays traditionally acknowledged therapeutic characteristics is a tuberous root, called by natives of Andean highlands “hipocotíleo”, although, depending on the reference source, subterraneous edible part of Maca is called hypocotyl or root and it is a common practice to describe Maca products being derived from Maca root(s), the term also adopted by Chacon (13, 21).

When describing cultivated Maca as a food plant of Peru, Leon (34), to its fleshy underground part of functional and therapeutic interest, used term “root-hypocotyl” (a fused secondary parenhymatic growth of hypocotyl tissue and the upper part of the taproot). According to Essig (16), a specialized taproot, or sometimes a hypocotyl swells up with food reserves, resulting in a carrot, radish, beet, parsnip, turnip, or one of many other type of root vegetable. The hypocotyl is the usually inconspicuous section of stem between the root proper and the seedling leaves (cotyledons) that mark the beginning of the leafy shoot and in biennials, such as radishes, this tiny section of stem swells into the underground storage organ, not the root proper. Therefore, taking into account the above botanical characteristics and visual appearance of both species (Figure 1), it was reasonable to accept the term hypocotyl for the underground (or subterranean) storage organ of cultivated Peruvian Maca labelled *L. peruvianum* and use the term root (root proper) in reference to the underground part of *L. meyenii.* The term “Maca hypocotyl” has been adopted and consistently used in many Maca-related publications (4, 7, 35), although Hermann and Bernet (33) are of the opinion, that the term hypocotyl in relation to Maca is misleading and its use should be discontinued. They (33) also acknowledged, that it is an important issue to find an appropriate name for underground part of Maca and then, consistently adhering to the use of proper term for this part of Maca plant, since it has implications in categorization, the key wording of literature, the assignation of tariff codes, etc. – which, apart from logistics view point, is not a trivial or irrelevant matter.

### Phytochemical differences between *L. meyenii* and *L. peruvianum*

The aerial part of Maca (consisting mostly leaves), contained no Glucosinolates but typically contain flavonoids which are used by plants to adapt to light conditions. Plants of the same genus can be expected to have more secondary metabolite compositional commonality than plants of a different genus. In the case of *Lepidium* the commonly reported flavonoids are Kaempferol derivatives, hence, they have been utilised as a marker which would help in distinguishing differences between *L. meyenii* and *L. peruvianum*. On examination of the MS spectra of both specimens it was evident, that they contained mixtures of both quercetin and kaempferol. Maca sample labelled as *Lepidium peruvianum* shared substantial commonality with the voucher specimen deposited under the same name in the SCU Herbarium of Medicinal Plants. The presence of distinctly different flavonoid derivatives (13–17 mins), which have not as yet been described, indicates an existence of species level differentiation between *L. meyenii* and *L. peruvianum*. MS fragmentation spectra of those compounds are consistent with kaempferol and quercetin diglycosides (Figure 3b), which have been also detected in *Lepidium perfoliatum* (36). Some commonality of the *Lepidium meyenii* Walpers with the specimen labelled *L. peruvianum* Chacon (Figure 3a) would be expected from two plants of the same genus. However, *L. meyenii* contains molecules which are distinctly extraneous to *L. peruvianum* (Figure 3b, 4 and 5) and may suggest a proposition that *L. meyenii* could be considered a different species - contrary to accepted description of Peruvian Maca under the two scientific names classified by Regulatory Agencies in the EU (37) and Australia (38, 39) as “*synonymous*”.

**Figure 10:**
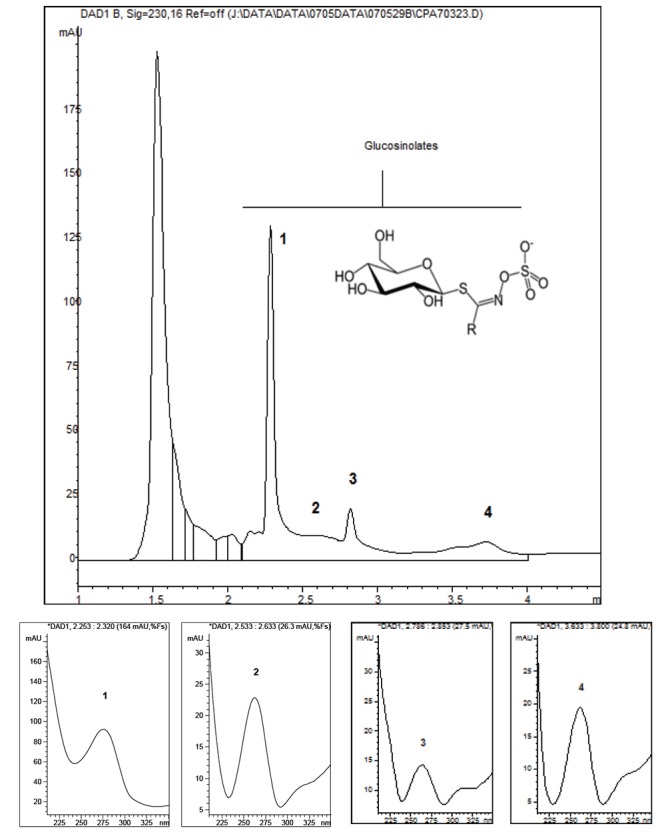
HPLC profile of Peruvian Maca (*L. peruvianum*) glucosinolates at 230 nm. Boxes 1 to 4 represent UV spectra of glucosinolates as identified on the HPLC profile measured as glucosinolates between 250-275 nm. Insert on the HPLC profile shows skeletal chemical structure of Glucosinolate (Side group R varies – depending on intermediary compound formed as secondary metabolite).

Subterranean part: On the basis of survey conducted by Li *et al.* (40) involving various Maca hypocotyls and products derived from cultivated Peruvian Maca – *L. peruvianum*, the most abundant Glucosinolates detected in fresh and dry material were the aromatic glucosinolates, benzylglucosinolyte (glucotropaeolin) and p-methoxybenzylglucosinolate. Therefore, it is reasonable to assume, that the distinctive major peaks on HPLC trace for the hypocotyl of *L. peruvianum* may be assigned to those compounds. The HPLC trace for subterranean part of *L. meyenii* has essentially different profile from the one of *L. peruvianum* in terms of shape and retention time for its key detected components (Figure 10). The observed differences in shapes of HPLC traces and lack of common peaks on HPLC resolution printouts for subterranean parts of *L. peruvianum* and *L. meyenii* (Figure 7a and 7b) demonstrate that they do not share common character, which would justify for both of these plants use the term “*synonymous*”.

Similarly to the 2D HPLC resolution profiles, the 3D spheroids confirmed observation that the qualitative glucosinolates profiles of the two isotypes of *L. peruvianum* and *L. meyenii* do not share commonality in terms of Glucosinolates profiles for the subterranean parts.

An exploratory attempt - although imperfect due to a single resolution run (only a small sample from the isotype of dried *L. meyenii* root deposited in Herbarium was made available for the test), it is worthwhile to demonstrate an appearance of different bands (with distinctive-smears) representing the two isotypes,which may indicate an existence of genetic differences and genetic polymorphism. Distinctive “smears” of DNA (Figure 9) from dry plant material confirmed that there were DNA segments of many lengths with small fragments which could be due to degradation in hypocotyls during dehydration process under the direct sun at the elevation of above 4,000 m above the see level, where UV light spectrum is extremely active (41). Intensive UV and extreme hard environmental conditions may induce mutations of certain genes to help adapt to the environment (42). This may also involve the change between diploid an amphiploid status of plant, thus increasing adaptation to the existing external conditions where plant is grown (4).

Further tests are required to accurately determine precise genetic differences between the two isotypes, preferably using freshly harvested roots and hypocotyls and more accurate techniques for resolution of DNA segments used, i.e. the Internal Transcribed Spacer Region of 18S-25S rDNA, so as to improve separation of bands distinctive to each Maca isotype.

In the meantime, the question remains however, as to validity of conflicting use of different scientific terminology attached to the common name Peruvian Maca, which continue to exist in both research literature and legislative terminology. Whether both isotypes discussed in this paper represent the same species under two alternative names, or are different species – this would require definite research confirmation, formal acknowledgement and corresponding correction in formal and practical terms.

### Two scientific names for Peruvian Maca – reasons for concern

Depending on the reference source, Maca of Peruvian origin continues being labelled under either of the two scientific names: as *Lepidium meyenii* Walpers or *Lepidium peruvianum* Chacon. The source of the confusion may be traced back to 1843, when a plant called by Bolivian natives “Maca” was collected in Pisacoma, the Department of Puno by German botanist Gerhard Walpers, who, on his exploratory mission in Peruvian-Bolivian part of the South American continent deposited a specimen of this plant as Maca in the USM Herbarium in Lima under taxonomic denomination *Lepidium meyenii* Walpers.

It was not until the early 1960s that Maca and its historically-unique properties started to be researched by Peruvian scientists Dr Gloria Chacon Realdon (14), who is credited with re-discovery of long forgot plant and acknowledged as the first scientist pioneering in-depth research into this ancient medicinal plant of Incas. Apparently, at the time when Dr Chacon started her work on Peruvian Maca, it has been formally known under scientific name *Lepidium meyenii* Walpers and in her first research work, published back in 1961, she has also referred to the herb Maca under it scientific name known at that time as *L. meyenii*.

The group of plants listed under genus *Lepidium* belonging to the *Brassicaceae* family is grown and distributed throughout the world in all continents (with exception of Antarctica). It appears that subterranean part of cultivated Peruvian Maca, referred to in research publications under the two scientific terms – *L. peruvianum* and *L. meyenii*, is the only plant from *Lepidium* genus, which is confirmed in analytical and clinical work, as displaying wide range of therapeutic properties. It contains the nutrients and other active compounds which appear to be responsible for traditionally known since the times of Incas, medicinal and beneficial health effects linked to the use of this herb by natives of Peru and users in other countries throughout the world, where the herb is exported (5-7, 9, 10, 22-24).

The distinctive edible hypocotyl present in *L. peruvianum,* conforms to the range of phenotypic and morphologic variability as outlined by Lebeda *et al*. (30), expected to exists among genotypes in harvested cultivated Maca crops (Figure 2). On the other hand, *L. meyenii* with its thin root, without any tuber-like storage portion along the root’s shaft, does not share phenotypic characteristics typically-observed in hypocotyls of cultivated Maca harvested in Andean highlands. The above confirms observation made back in 1945 by Weberbauer (43), describing *Lepidium meyenii* Walpers as high plateau grass without clearly developed hypocotyl, growing at above 4000 meters a.s.l. Visual and microscopic comparison of holotypes and isotypes of *L. meyenii* and *L. peruvianum* conducted by Chacon (5), showed dissimilarity of the type specimen with the cultigen in terms of the absence of a pronouncedly tuberous root and the state of domestication, what led to her conclusion, that these two isotypes represent different species, with *L. peruvianum* conforming in morphology terms to cultivated Maca.

In 2005, the Panel of Peruvian Experts (*Plant Taxonomy Specialists’ Work Group Regarding the Botanical Name of Maca*) started an investigative procedure to clarify the issue of an error in the scientific name used for Maca and some 3 years later, adopted *Lepidium peruvianum* Chacon as the scientific name representing cultivated Peruvian Maca, with official publication in Peruvian Normas Legales-375606 (44). The above considerations regarding scientific labelling of cultivated Maca as *L. peruvianum* have found support in an annotation published back in 1996, added in a proof stage of the paper by Quiros *at al.* (4):

*“Comparison of Maca with specimens of L. meyenii deposited at the herbaria in UC Berkeley, Frei U. of Berlin, Javier Prado Museum in Lima and Cesar Vergas Herbarium in Cuzco, Peru, fails to demonstrate morphological similarities. Furthermore, the existing L. meyenii accessions were collected outside the range of distribution of the cultivated species. Therefore, renaming the cultivated species as L. peruvianum Chacon is justifiable.”*

In 2007, Hermann and Bernet (33) in their elegant monograph on the transition of Maca from neglect to market prominence provided a reasonably objective account of Maca as a crop and in-depth analysis of Maca in biological, agricultural and marketing context. In their opinion, reasons stated by Chacon in justifying her decision to give cultivated Maca species status are not consistent with modern taxonomic and nomenclatural practice, since a type specimen used in the description of the plant, may represent a less common variant. Also, the status of domestication or the size of a vegetative plant part in an intra-specific population is considered today as insufficient for the description of a new species. In the case of cultivated Maca, wide variation in size and shape of roots in harvested crop in which, as per observation cited by the authors (33), that up to 20% of plants may have no tuberous roots at all, did not present evidence for discontinuous variation or reproductive barriers between wild and domesticated forms of Maca.

Standardisation of scientific name for the cultivated Peruvian Maca would eventually help in gradual elimination of unintentional taxonomic error introduced by Walpers, which continues to be made until today by numerous authors and institutions using the scientific name *Lepidium meyenii*. It is believed, that Mr Walpers – if he would be alive today - would not object to such analytically supported taxonomic clarification in scientific terminology, differentiating the cultivated Maca herb *L. peruvianum* from a wild grown Maca plant *L. meyenii* collected by him back in 1843, when presently used research tools and analytical techniques were not available in his days.

It appears that, irrespective the formal adoption of the *L. peruvianum* as the scientific name for cultivated Peruvian Maca (44), its old scientific name given by Walpers is still the only name formally recognized by the USDA (12). Here it is worth to add, that Hermann and Bernet (33) in their monograph mentioned about an influential consortium of Peruvian Maca Companies linked to the US-based shareholding interest (where Maca has been patented under the name of *L. meyenii*) and who have been accused of improper motives, recommend and defend continued use of the name *L. meyenii* as the valid name for Maca in the USA. The ongoing public debate over the ‘correct’ botanical name for Maca is an interesting case where taxonomic and nomenclatural disagreements get in the way of the functioning of supply chains.

In the EU 2007 Catalogue (37) both names *L. meyenii* and *L. peruvianum* are currently accepted for use as “*synonymous”*. In Australia, Food Standards Australia and New Zealand (FSANZ) accepts the use of Maca powder as non-traditional and not novel food under the scientific name *Lepidium meyenii,* although *Lepidium peruvianum* is considered as the synonym (38). In 2006, the Therapeutic Goods Administration (TGA) acting on the advice from Medsafe Interim Joint Expert Advisory Committee on Complementary Medicines (IJEACCM), considered a *Lepidium meyenii* preparation consisting of the dried, powdered, uncooked tuberous root as suitable for use in ‘Class 1 (low risk)’ medicines, without restriction (39). The name “*Lepidium meyenii*” is also accepted by the ACCM (39) as an Australian Herbal Name (AHN) with synonym: *Lepidium peruvianum* Chacon for Maca plant used as traditional Peruvian food. However, on the advice from the ACCM, TGA does not accept *L. meyenii* and *L. peruvianum* as separate species, due to the fact that the data provided to the ACCM are still inadequate to support the safety and quality of the *L. meyenii* as a new complementary medicine substance for use in listed medicines in Australia (39).

In China, after initial approval for import of Maca products to China in 2002, Chinese National Ministry of Health has approved the use of Maca powder in 2011 as the new resource food under the scientific name *L. meyenii* (45). However, it is not clear whether *L. meyenii* is referred to Maca plant of Peruvian origin grown in Andean Highlands or to “Chinese Maca” cultivated in Qinghai-Tibet Plateau, Yunnan, where Maca plantations were apparently established in China back in 2003.

It is reasonable to assume, that as with most changes, it will take time for academics, commerce, relevant Government and international Legislative Bodies to adopt correct scientific name *Lepidium peruvianum* Chacon for cultivated Peruvian Maca – as the only plant, which was verified in clinical trials, as capable to induce its traditional functionalities for expected health benefits.

## CONCLUSIONS

Taking into account botanic, taxonomic and biochemical facts presented in this paper, it is concluded that the scientific name *Lepidium peruvianum* Chacon should be reserved exclusively to cultivated Peruvian Maca plants with their distinctively-formed subterranean part tuberous roots - hypocotyls utilised for food and/or dietary supplement.

The conclusion as presented in this paper is in line with a consensus of opinion reached by the panel of Peruvian scientific specialists and experts in taxonomy on Maca and other native plant species, who have corrected scientific name for cultivated Peruvian Maca, being now formally referred to by Peruvian Normas Legales 375606 as *Lepidium peruvianum* Chacon.

The question still remains as to validity of conflicting international use of different scientific terminology attached to the common name “cultivated Peruvian Maca”, whether both isotypes represent different species under two alternative names and if yes - then, the use of the term “*synonymous*” should be reviewed for further definite research confirmation, formal acknowledgement and – if justified - corresponding correction in formal and practical terms internationally.

## ABBREVIATIONS

ACCM: Advisory Committee on Complementary Medicines
ACN: Acetonitrile
APCI: Atmospheric Pressure Chemical Ionisation
a.s.l.: above sea level
CA, USA: State of California, United States of America
DNA: Deoxyribonucleic acid
EI: Extracted Ion
EIC: Extracted Ion Chromatogram
EU: European Union
HPLC: High Performance Liquid Chromatograph
IJEACCM: Interim Joint Expert Advisory Committee on Complementary Medicines
KH_2_PO_4_: Potassium Dihydrogen Phosphate
LCMS: Liquid Chromatograph Mass Spectrometer
MS: Mass Spectrometer
MS-TIC: Mass Spectrum – Total Ion Chromatogram
OCM: Office of Complementary Medicines
PDA: Photo Diode Array
RIMP: Research Institute of Medicinal Plants, Poznan, Poland
RMIT: Royal Melbourne Institute of Technology
SCU: Southern Cross University, Lismore, Australia
SMU: San Marcos University in Lima
TFA: Triflouroacetic acid
TGA: Australian Therapeutic Goods Administration
USDA: United States Department of Agriculture

## References

[1] Pulgar VJ (1960). La Maca (*Lepidium sp.*) poderoso fecundate vegetal. La Voz de Huanaco.

[2] Johns T (1981). The Anu and the Maca. J. Ethnobiology.

[3] Tello J, Hermann M, Calderon A (1992). La maca (Lepidium meyenii Walp): cultivo alimenticio potencial para las zonas Altoandinas. Bot. Lima.

[4] Quiros CF, Epperson A, Hu J, Holle M (1996). Physiological Studies and Determination of Chromosome Number in Maca, Lepidium meyenii (Brassicaceae). Economic Botany.

[5] (2001). Chacon Gloria de Popovici. Maca (*Lepidium peruvianum* Chacon) Planta Millenaria del Peru, con Propiedades Altamente Nutricionaly Medicinal. Lima, Peru.

[6] Obregon LV (2001). “Maca” Planta Medicinal y Nutritiva del Peru.

[7] Gonzales GF (2012). Ethnobiology and Ethnopharmacology of *Lepidium meyenii* (Maca), a Plant from the Peruvian Highlands [Review article on the Internet]. Evid Based Complement Alternat Med. [Article ID 193496].

[8] Gonzales GF, Gasco M, Lozada-Requena I (2013). Role of maca (Lepidium meyenii) consumption on serum interleukin-6 levels and health status in populations living in the Peruvian Central Andes over 4000 m of altitude. Plant Foods Hum Nutr.

[9] Gonzales GF, Villaorduña L, Gasco M (2014). Maca (Lepidium meyenii Walp), a review of its biological properties. Rev. Peru Med. Exp. Salud Publica.

[10] Meissner HO, Kapczynski W, Mscisz A (2015). Use of Gelatinised Maca (*Lepidium peruvianum*) in Early-Postmenopausal Women - a Pilot Study. Intnl Journ. Biomed. Sc.

[11] Meissner HO, Reich-Bilinska H, Mscisz A (2006). Therapeutic effect of pre-gelatinised Maca (*Lepidium Peruvianum* Chacon) used as non-hormonal alternative to HRT in peri-menopausal women. Intnl Journ. Biomed. Sci.

[12] USDA PLANTS United States Department of Agriculture, Natural Resources Conservation Service. Lepidium meyenii Walp.

[13] Chacón Roldan G (1960). Comunication sobre un *Lepidium sp.* Publicacion de las Catedras de Quimica Organica y Quimica Aplicada a las Ciencias Biologicas, Facultad de Ciencias de la Univ. Nac. San Marcos, Lima, Peru.

[14] (1990). Holotypus *Lepidium peruvianum* Chacon. No 89129 USM.

[15] Chacón de Popovici G (1990). La Maca (Lepidium peruvianum Chacón sp. nov.) y su Hábitat. Revista Peruana de Biología.

[16] Essig FB (2012). Plant Life. [Monograph on the Internet]. Oxford University Press.

[17] Sandoval M, Okuhama NN, Angeles FM (2002). Antioxidant activity of the cruciferous vegetable Maca (*Lepidium meyenii*). Food Chemistry.

[18] Tellez MR, Khan IA, Konaisy M (2002). Composition of the essential oil of *Lepidium meyenii* (Walp). Phytochemistry.

[19] Fahey JW, Zalcmann AT, Talalay P (2001). The chemical diversity and distribution of glucosinolates and isothiocyanates among plants. Phytochemistry.

[20] Valerio L, Gonzales GF (2005). Toxicological aspects of south American herbs: *Uncaria tomentose* (Cat’s Claw) and *Lepidium meyenii* (Maca). A Critical Synopsis. Toxicological reviews.

[21] Chacón Roldan G (1961). Estudio fitoquimico de *Lepidium meyenii* Walp. Thesis “*Phytochemical Studies of Lepidium meyenii Walp*”. Lima, Peru: Univ. Nac. San Marcos.

[22] Dini A, Migliuolo L, Rastrelli P (1994). Chemical composition of *Lepidium meyenii*. Food Chemistry.

[23] Clément C, Diaz Grados DA, Avula B (2010). Influence of colour type and previous cultivation on secondary metabolites in hypocotyls and leaves of maca (*Lepidium meyenii* Walpers). J. Sci. Food Agric.

[24] Meissner HO, Mscisz A, Reich-Bilinska H (2006). Hormone-Balancing Effect of Pre-Gelatinized Organic Maca (Lepidium peruvianum Chacon): (II) Physiological and symptomatic responses of early-postmenopausal women to standardized doses of Maca in Double Blind, Randomized, Placebo-Controlled, Multi-Centre Clinical Study. Intnl Journ. Biomed. Sci.

[25] Piacente S (2002). Investigation of the Tuber Constituents of Maca (Lepidium meyenii Walp.). J. Agric. & Food Chem.

[26] McClure K (2004). The Biological Effects of Maca. BNat (Hons) Thesis, Southern Cross University.

[27] ARL-TM125 (2014). Analytical Laboratory Procedure, Technical Protocol 125.

[28] Bon MC, Hurard C, Gaskin J, Risterucci AM (2005). Polymorphic microsatellite markers in polyploid Lepidium draba L. ssp. draba (Brassicaceae) and cross-species amplification in closely related taxa. Molecular Ecology Notes.

[29] Echegaray MP (1999). Caracterizacion agronomica y seleccion de differentes morfotipos de maca (Lepidium sp.) faze vegetative, en su habitat natural.. Curso Tallar Internacional sobre Maca “Cultivo, aprovechamiento y conservacion”.

[30] Lebeda A, Dolezalova I, Valentova K (2003). Biological and Chemical Variability of Maca and Yakon. Chem. Listy.

[31] (1999). Ministerio De Agricultura Del Peru. Proyecto Nacional De Manejo De Cuencas Hidrorafias Y Conservacion De Suelos. Pronamachcs. Direccion Departamental Junin. Agencia Junin. Maca. (Lepidium meyenii Walpers).

[32] Brinckmann J, Smith E (2004). Maca Culture of the Junín Plateau. J. Altern. Complement Med.

[33] Hermann M, Bernet T (2009). The transition of maca from neglect to market prominence: Lessons for improving use strategies and market chains of minor crops. Agricultural Biodiversity and Livelihoods. Discussion Papers 1.

[34] León J (1964). The Maca (*Lepidium meyenii*) a little-know food plant of Perú. Economic Botany.

[35] Ganzera M, Zhao J, Muhammad I, Khan IA (2002). Chemical Profiling and Standardistaion of *Lepidium meyenii* (Maca) by Reversed Phase High Performance Liquid Chromatography. Chemical and Pharmaceutical Bulletin.

[36] Fursa NS, Litvinenko VI (1970). Lepidoside from Lepidium perfoliatum. Chemistry of Natural Compounds.

[37] (2007). The EU Catalogue on Botanicas and Various Ingredients. Summary of reported facts until CAFAB-32 & new items for CAFAB-33/Guido Mattera Ricigliano; Maca–EU Green List Status, Regulation 258/97/EC, #134; 30/51.

[38] Food Standards Australia New Zealand. [database on the Internet]. Record of views formed by the FSANZ Novel Foods Reference Group or the Advisory Committee on Novel Foods. [database on the Internet]. Record of views formed in response to inquiries (Updated August 2014). http://www.foodstandards.gov.au/Search/pages/results.aspx?k=maca%20lepidium%20meyenii.

[39] ACCM Advisory Committee on Complementary Medicines. [database on the Internet]. 5.1 Lepidium meyenii. https://www.tga.gov.au/committee-meeting-info/accm-extracted-ratified-minutes-meeting-7-2-september-2011.

[40] Li G, Ammermann U, Quiros QF (2001). Glucosinolate contents in Maca (*Lepidium peruvianum* Chacon) Seeds, Sprouts, Mature plants and Several Derived Commercial Products. Economic Botany.

[41] (2006). Arroniz-Crespo. Physiological Changes and UV protection in the aquatic liverwort *Jungermannia exsertifolia* subsp. *Cordifolia* along an altitudinal gradient of UV-B radiation. Functional Plant Biology.

[42] Ryan K (2002). Flavonoid Gene Expression and UV Photoprotection in transgenic and mutant Petunia leaves. Phytochemistry.

[43] Weberbauer A (1945). El Mundo Vegetal de los Andes Peruanos.

[44] (2008). Normas Legales. Peruvian Experts’ Panel. Plant Taxonomy Specialists’ Work Group Regarding the Botanical Name of Maca., Peruvian legislation No. 375606. Lima, Peru.

[45] CN 102429066 A. [database on the Internet]. Physical strength-enhancing anti-fatigue compound Maca coffee product. http://www.google.com/patents/CN102429066A?cl=en#legal-events.

